# Physicochemical Interactions in Nanofunctionalized Alginate/GelMA IPN Hydrogels

**DOI:** 10.3390/nano11092256

**Published:** 2021-08-31

**Authors:** Rana Kadri, Kamil Elkhoury, Ghazi Ben Messaoud, Cyril Kahn, Ali Tamayol, Joao F. Mano, Elmira Arab-Tehrany, Laura Sánchez-González

**Affiliations:** 1 LIBio, Université de Lorraine, F-54000 Nancy, France; rana.kadri@hotmail.com (R.K.); kamil.elkhoury@univ-lorraine.fr (K.E.); ghazi.benmessaoud@hotmail.fr (G.B.M.); cyril.kahn@univ-lorraine.fr (C.K.); 2 Department of Biomedical Engineering, University of Connecticut, Mansfield, CT 06269, USA; atamayol@uchc.edu; 3 Department of Chemistry, CICECO—Aveiro Institute of Materials, University of Aveiro, 3810-193 Aveiro, Portugal; jmano@ua.pt

**Keywords:** alginate, gelatin, IPN hydrogels, functionalization, nanoliposomes, biomimetic nanocomposites

## Abstract

Polymeric hydrogels are currently at the center of research due to their particular characteristics. They have tunable physical, chemical, and biological properties making them a material of choice for a large range of applications. Polymer-composite and nanocomposite hydrogels were developed to enhance the native hydrogel’s properties and to include numerous functionalities. In this work, alginate/gelatin-methacryloyl-based interpenetrating polymer network hydrogels were prepared with different alginate concentrations and investigated before and after the functionalization with nanoliposomes. The multiscale analysis was obtained through Fourier transform infrared spectroscopy and proton nuclear magnetic resonance. The results show interactions between two polymers as well as between the nanoliposomes and biopolymer.

## 1. Introduction

Hydrogels are receiving considerable interest and are becoming more widespread because of their potential applications, especially in food, tissue engineering, cosmetics, and drug delivery. In the presence of an aqueous-based solvent, hydrogels swell and absorb a high amount of water due to the hydrophilic character of the constituents of the three-dimensional network. They are prevented from dissolving and can maintain their shapes due to the physical or chemical crosslinking within the chains. The swelling properties provide hydrogels with a significant degree of flexibility and soft consistency.

Various types of polymers, including natural, synthetic, and semi-natural polymers, have been used for hydrogels formation, defining the characteristics of the network [1]. The synthetic polymers present networks with strong mechanical characteristics and grant better control over the properties. Natural polymers have outstanding characteristics for biological applications such as biocompatibility and often enzyme-driven degradability [2].

Among the most studied natural polymers, alginate, a natural polysaccharide extracted from algae or bacterial biofilm, is well-known due to its widespread applications. It presents remarkable properties such as biocompatibility, non-immunogenicity, and low toxicity [3,4,5,6]. Alginate hydrogels are physically crosslinked in the presence of divalent cations, such as calcium [7]. Two units of two different chains of alginate bind with the same calcium and form a junction. This association results in the formation of an “egg-box”, which eventually forms the network.

Another interesting polymer is gelatin methacryloyl (GelMA). It is the result of the modification of gelatin, a natural and cytocompatible protein. Gelatin is an interesting polymer due to the presence of Arg-Gly-Asp (RGD), bioactive sequences that are effective in cell entrapment and allow active molecules to bind to the polymeric network [8]. It is a semi-natural polymer derived from the substitution of the amine group of gelatin with methacrylate anhydride [9]. GelMA is a photocrosslinkable hydrogel, which, once exposed to UV light, forms a covalent bond [10]. The crosslinking process requires the addition of a photoinitiator that breaks down into radicals in the presence of UV irradiation. In addition to UV light, the two-photon polymerization method can be used to crosslink GelMA [11]. The objective of the gelatin modification is to obtain an irreversible and stable hydrogel with temperature variations.

There have been many important advances in the field of materials to develop potentially applicable hydrogels to be utilized in various domains. New complex networks with valuable properties are required to overcome the limitations and challenges of using single polymer formulations. The reinforced composite hydrogels resulting from the incorporation of other entities, namely nanofibers or nanoparticles, permit the enhancement and optimization of the network properties and expand their field of use [12,13].

Polymer composite hydrogels are hydrogels formed by the mixture of several polymers with different characteristics. Polymer composite hydrogels can form an interpenetrating polymer network (IPN) when the added polymer forms a secondary network within the formed hydrogel matrices. Due to the combination of the polymers, an advanced multicomponent polymeric system is formed [14]. IPN hydrogels combine the advantageous characteristics of each polymer component [15]. For example, alginate alone presents poor cell bonding properties due to the absence of cell attachment ligands and low protein adsorption [16]. However, arginine–glycine–aspartic acid (RGD) sequences that are beneficial for cell adhesion are abundant in gelatin [17]. So, instead of coupling RGD-containing peptides to the alginate backbone, IPN hydrogels with improved biocompatibility combining GelMA and alginate can be easily formed.

Nanocomposite hydrogels are obtained by incorporating nanoparticles in the polymeric network. This combination can add advantageous chemical, physical, and biological properties and provide superior functionality to an effective application of hydrogels in many fields [18,19,20,21]. Nanoparticles can enhance the transport and the release of active molecules and preserve them from premature degradation or deterioration [22].

Nanoliposomes are soft and natural nanoparticles, composed essentially of phospholipids [23,24,25]. They are negatively charged amphiphilic molecules, organized in a bilayer with hydrophobic tails in the center and polar hydrophilic heads facing the solution [26,27]. This particular character makes liposomes an effective system for the delivery of different types of encapsulated molecules [28]. A powerful advantage of liposomes is their ability to transport and release both hydrophilic and hydrophobic bioactive molecules. An advanced, sustained, and controlled drug delivery system can be created by functionalizing the IPN hydrogels with nanoliposomes. Rapeseed nanoliposomes-functionalized IPN alginate/GelMA hydrogels have the potential to be used as tissue engineering scaffolds [29,30].

The purpose of this study was to form IPN hydrogels with the combination of alginate and GelMA and to study the possible interactions occurring in the 3D network. Additionally, rapeseed nanoliposomes were incorporated in the hydrogels to analyze the interaction of the polymers with the soft nanoparticles. The physicochemical characterization of this nanofunctionalized system has not yet been performed. This characterization helps with unveiling interactions in the 3D network, which better highlights potential future applications of IPN alginate/GelMA hydrogels nanofunctionalized with rapeseed nanoliposomes.

## 2. Materials and Methods

Alginic acid sodium salt (SA) from brown algae (M/G ≈ 1.56) with an average viscosity molecular weight M*_v_* of 1.69 × 10^5^ g mol^−1^ according to the correlation of Mark–Houwink–Sakurada: $[\eta ]=KM_{v}^{\alpha }$ where α = 0.92 and k = 7.3 ×10^−5^. Calcium chloride dehydrate, used for alginate crosslinking, was provided by VWR (International, Leuven, Belgium). Gelatin (type A, 300 bloom from porcine skin), methacrylic anhydride (MA), photoinitiator (PI) 2-hydroxy-4′-(2-hydroxyethoxy)-2-methylpropiophenone, and phosphate-buffered saline (PBS) tablets were purchased from Sigma-Aldrich (Chemie, Steinheim, Germany). Rapeseed lecithin was provided by Solae Europe SA society (Geneva, Switzerland).

### 2.1. Synthesis of Alginate Hydrogel

Alginate acid sodium salt was dissolved in double-distilled water with a concentration of 2% (*m*/*v*). Once the solution was homogeneous, 2 mL of the solution was transferred slowly using a pipette to a petri dish containing 5 mL of CaCl_2_. The reaction of alginate with calcium was left for 24 h at 4 °C to achieve a crosslinked alginate hydrogel.

### 2.2. Synthesis of GelMA Hydrogel

A total of 10% wt of pork skin gelatin type A was dissolved in PBS at 60 °C. We then added 8 mL of methacrylate to the solution to substitute the free amine groups of the gelatin with the methacrylic anhydride. After 3 h of stirring, 400 mL of PBS (60 °C) was added to the solution. Diluted GelMA was dialyzed at 40–50 °C using a dialysis membrane (Spectro/Por molecular porous membrane tubing, MWCO 12–14,000, Fisher Scientific, Hampton, NH, USA) and then lyophilized for 1 week.

The freeze-dried powder GelMA (30%, *m*/*v*) was melted in PBS at 40 °C. To absorb the UV light during the gelation, 1% of photoinitiator 2-hydroxy-4′-(2-hydroxyethoxy)-2-methylpropiophenone was added to the solution at 80 °C. An amount of 1 mL of GelMA was then exposed to UV light (360–480 nm) for 240 s.

### 2.3. Polymer-Composite Hydrogel Synthesis

The alginate/GelMA solution was prepared at 40 °C by mixing 3 different concentrations of alginate (0.5%, 1% and 2%, *m*/*v*) with GelMA (30%, *m*/*v*). An amount of 1 % (*m*/*v*) of PI was added to the final blended solution. The crosslinking started by spreading 2 mL of the mixture in 5 mL of CaCl_2_ solution (2%, *m*/*v*). This first step permitted the crosslinking of alginate. Then, the semi-crosslinked hydrogel was exposed to UV light for 240 s to allow the free radicals photopolymerization of the GelMA.

### 2.4. Liposomes Preparation

Two different concentrations, 3% and 5% (*w*/*w*), of rapeseed lecithin were dissolved in double-distilled water and stirred at inert atmosphere (nitrogen). The solution was then sonicated at 40 kHz and 40% of full power for 300 s (1 s on/1 s off) to homogenize the solution. The liposomal suspension was kept from the light at 4 °C. The solution could be conserved for up to one month. The nanoliposomes were then filtered using sterile syringe filters with a 0.22 µm pore size and added to each solution with a concentration of 8.6% (*v*/*v*) before the gelation process.

### 2.5. Characterization of Hydrogels

#### 2.5.1. Zeta Potential Measurements

The electrophoretic mobility was measured for the different solutions before the gelation to determine the net charge of the polymers and the nanoparticles. The measurements were recorded using a Malvern ZetasizerNAno ZS (Malvern Instruments, Worcestershire, UK) with DTS Nano software (6.12, Malvern Instruments, Worcestershire, UK). The samples were diluted (1:2) to obtain better resolution and placed in standard capillary electrophoresis cells equipped with gold electrodes. All measurements were carried out at 37 °C. The presented results are the mean of three measurements.

#### 2.5.2. Fourier Transform Infrared Spectroscopy

The FTIR spectra of freeze-dried hydrogels were recorded with a Tensor 27 mid-FTIR Bruker spectrometer (Bruker, Karlsruhe, Germany) equipped with an ATR accessory. A total of 128 scans were used for both reference and samples between 4000 and 400 cm^−1^ at a 4 cm^−1^ resolution. Spectral manipulations were then performed using OPUS software (Bruker, Karlsruhe, Germany). Raw absorbance spectra were smoothed using the 9 points smoothing function. After elastic baseline correction using 200 points, H_2_O/CO_2_ correction was then applied. Then, spectra were centered and normalized. All tests were run in triplicate.

#### 2.5.3. ^1^H-NMR

The chemical modifications were also determined using proton nuclear magnetic resonance (^1^H-NMR) spectroscopy. The imaging was performed using Bruker Avance III 400 NMR spectrometer (Bruker, Karlsruhe, Germany). The polymers were dissolved, at the same concentration described above, in deuterium oxide (D_2_O) to stabilize the magnetic field and eliminate any perturbation in the spectrum. Hydrogels were introduced into 2 mm tubes and measured with a stable temperature of 37 °C. The results were recorded with a chemical shift in hydrogen ranging from −4 to 16 ppm and treated with an ACD/NMR processor program.

## 3. Results and Discussion

### 3.1. Zeta Potential Measurement

The zeta potential of hydrogels was measured, as it can quantify the charge repulsion/attraction or electrostatic magnitude between particles, which is known to affect stability. Alginate polymer contains carboxylic groups that are responsible for the negative charge of the polymer (Table 1). Liposomes are also negatively charged (−3.41 ± 0.05 mV) due to the presence of phospholipids. The isoelectric charge of GelMA, measured at pH 5, is −1.6 mV. The modification of gelatin with methacrylate anhydride leads to the replacement of the amine groups by carboxylic acid groups, producing a negative charge of the polymer. The addition of liposomes slightly affects the charge of GelMA (Table 1). GelMA has an amphoteric character and presents both negative and positive charges. The nanoliposomes added in a small quantity may react with the free amine groups of GelMA and their charges are neutralized. In the blended polymers system, the presence of alginate decreases the charge of GelMA to reach −21.83 mV. The presence of the positive charge of amine groups in GelMA allows the possibility for alginate polymer and nanoliposomes to interact with GelMA.

### 3.2. Fourier Transform Infrared Spectroscopy Analysis

**Figure 1:**
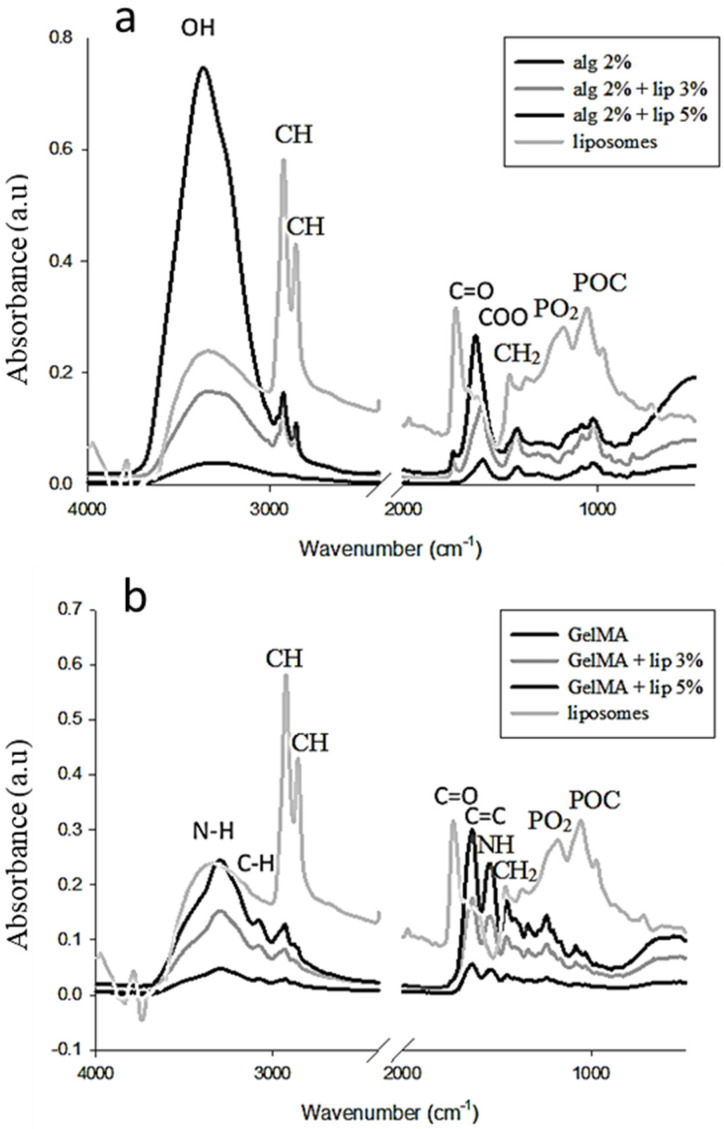
Surface analysis by FTIR of (**a**) alginate and (**b**) GelMA before and after functionalization with liposomes (3% and 5% (*w*/*v*)).

Fourier transform infrared (FTIR) spectroscopy, which is usually used to identify organic materials by measuring the absorption of infrared radiation by the sample material versus wavelength, was used here to characterize the presence of specific chemical groups in the hydrogels and to study the interaction between the blended polymers and the effect of the addition of the nanoliposomes in the polymers. Figure 1a shows the spectra of pure alginate hydrogel and alginate functionalized with nanoliposomes. The spectrum of alginate presents a peak at 1608 cm^−1^ due to the stretching of COO asymmetric elongation carboxylate indicating the content of uronic acid in the polymer. At 1415 cm^−1^, the peak is attributed to the symmetric stretching vibration of COO-. The CO stretching band appears at 1030 cm^−1^. The peak between 3250 and 3386 cm^−1^ represents OH stretching vibration. The stretching vibration of aliphatic CH appears at 2920–2850 cm^−1^ due to the addition of liposomes [31]. The FTIR spectrum of the alginate with nanoliposomes covers the peaks found in both alginate and liposomes spectra and does not show any interaction between the soft nanoparticles and the polymer. The peak at 1735 cm^−1^, which appears in the functionalized hydrogel, is assigned to the C=O stretching band of the liposomes. The absorbance intensity of the two peaks at 1620 and 3380 cm^−1^ increases with the addition of nanoliposomes in the alginate hydrogel. The increased percentage is relatively related to the amount of the added nanoparticles. The hydroxyl and carboxylate groups representing these two peaks are the responsible elements of the interaction of alginate with calcium. These elements present a narrow absorption region and the peaks are shifted to lower wavenumbers after the addition of the soft nanoparticles. The result confirms that the incorporation of nanoliposomes in the hydrogels affects the gelation process of alginate due to an interaction between the calcium and the nanoliposomes.

Figure 1b shows the spectra of the GelMA hydrogels derived from the modification of the gelatin with the methacrylate anhydride, with and without liposomes. A strong peak appears at 1650 cm^−1^ related to amide I primarily C=O stretching groups. The band at 1500–1570 cm^−1^ corresponds to C–N–H bending, while the band at 3200–3400 cm^−1^ indicates the presence of a peptide bond (mainly N–H stretching). The peak at 3062 cm^−1^ represents the C–H stretching groups [32].

GelMA presents a peak at 1640 cm^−1^ related to the carbon double bond that is present in the methacrylate, allowing the interaction between gelatin and methacrylate anhydride. The spectrum of the hydrogel incorporating nanoparticles does not show the peaks corresponding to the liposomes spectra, but it presents an increase in the intensity of certain peaks especially around 3300 cm^−1^. This result shows an interaction between the GelMA and the nanoparticles.

**Figure 2:**
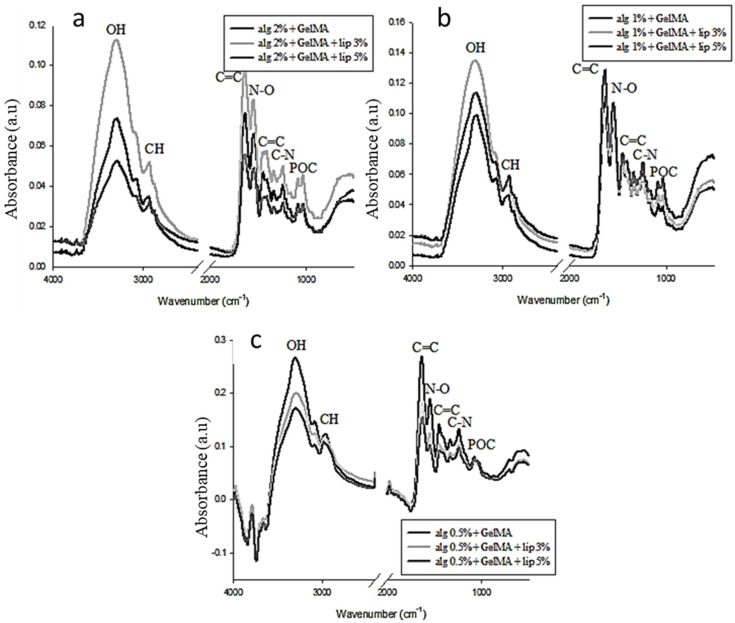
Surface analysis by FTIR of the mixture of GelMA and alginate with different concentrations of alginate. (**a**) Spectra of the mixtures with 2% (*m*/*v*) alginate, (**b**) spectra of the mixtures with 1% (*m*/*v*) alginate, and (**c**) spectra of the mixtures with 0.5% (*m*/*v*) alginate.

The FTIR spectra of the double network hydrogels (Figure 2) show new peaks, a sign of the presence of interconnection between the two networks. The alginate concentration has a considerable effect on the position of the peaks as well as their intensity.

The added nanoliposomes also react with the mixture of the polymers, and its impact on the hydrogel is influenced by the concentration of alginate. The presence of the alginate disturbs the normal interaction between GelMA and the nanoliposomes due to the interaction that occurs firstly between the two polymers.

### 3.3. ^1^H-NMR Characterization

**Figure 3:**
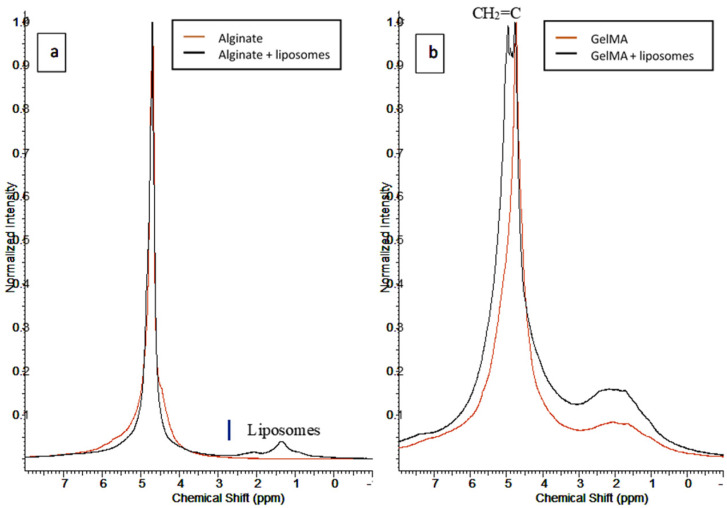
^1^HNMR spectra of (**a**) alginate and (**b**) GelMA before and after nanofunctionalization.

^1^H-NMR was used to determine the structure of hydrogels’ molecules by applying a nuclear magnetic resonance in NMR spectroscopy with respect to the ^1^H nuclei. Figure 3a shows the results of ^1^H-NMR obtained for alginate hydrogels, with and without nanoliposomes. These results present an interaction between the alginate polymer and the nanoliposomes, confirming the FTIR analysis. The spectrum shows a broad peak at 4.6–4.8 ppm representing the alginate polymer and the D_2_O solution in which it is solubilized. After the addition of liposomes, this peak does not change, while a new peak attributed to the liposomes appears at 1.35 ppm.

GelMA hydrogel (Figure 3b) shows a large peak at 1.1–2.9 ppm assigned to methyl and methylene residues of amino acids present in the hydrogel. The present peak also confirms the reaction between the –COOH group of the methacrylate anhydride with the –NH2 of lysine (1.61 and 2.93 ppm) in the gelatin. The addition of methacrylate in the gelatin provokes the formation of H_2_C=C(CH_3_)–in the solution, which leads to the apparition of two peaks at 5.64 and 5.36 ppm. The spectrum of GelMA does not show these peaks due to the polymerization of the double bonds during hydrogel formation.

**Figure 4:**
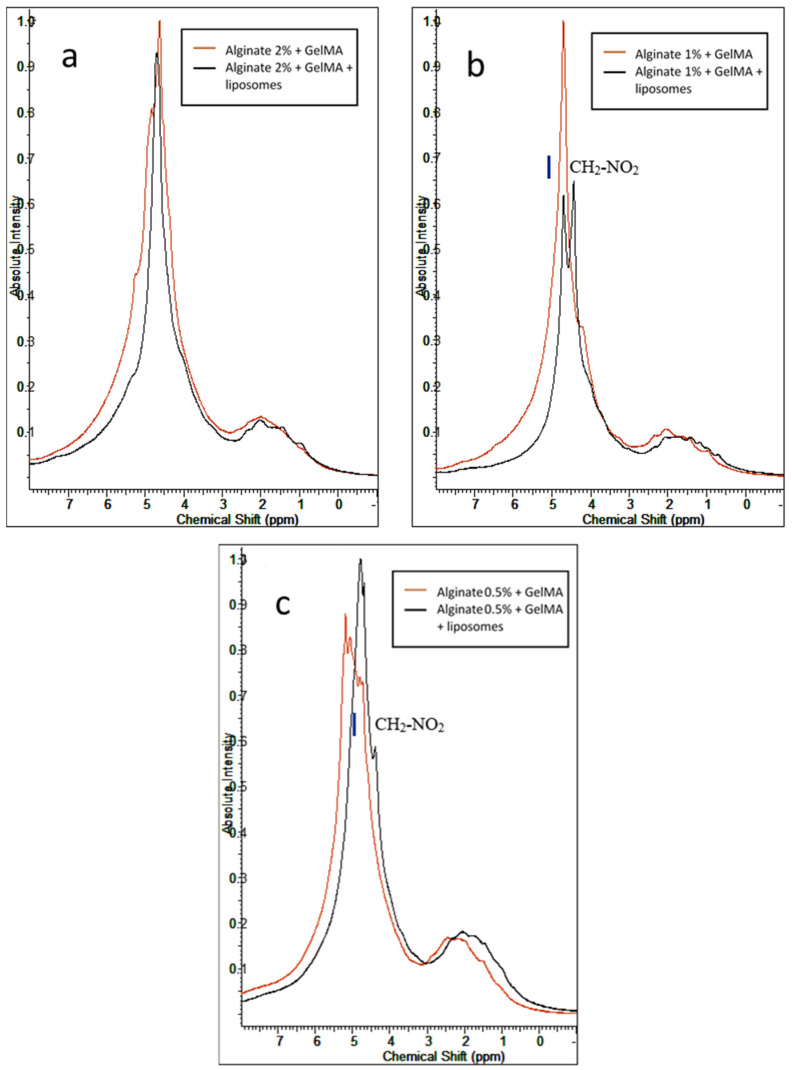
^1^HNMR spectra of the mixture between GelMA and alginate at different concentrations of alginate with and without nanoliposomes: (**a**) mixture with 2% alginate, (**b**) mixture with 1% alginate, and (**c**) mixture with 0.5% alginate.

The mixture of the two polymers (Figure 4) before the incorporation of nanoliposomes shows new peaks resulting from the electrostatic interaction between alginate and GelMA. GelMA is classified as an ampholytic polymer due to its composition in amino acids containing both negative and positive charges. The negatively charged alginate reacts with the positive charges present in GelMA, creating new links in the network and leading to the apparition of new peaks. The functionalization of the hydrogels with soft nanoparticles provokes the perturbation of the reticulation process of alginate and disrupts the formation of egg-box due to an electrostatic interaction between the liposomes and the crosslinking agent Ca^2+^. Liposomes can also, like alginate, interact with the positive charges present in the GelMA polymer. These interactions are responsible for the modification shown in the spectra of IPN hydrogels after the functionalization. The comparison of the spectra of the IPN hydrogels shows that an increase in the alginate concentration involves a predominance of the peak representing alginate and decreases the effect of liposomes in the hydrogels. A decrease in the concentration of alginate facilitates the reaction of the liposomes with GelMA.

Overall, the uncovered interactions between the IPN alginate/GelMA hydrogels and rapeseed nanoliposomes, coupled with the findings of previous work [29,30], suggest that the nanofunctionalization step reinforced its matrix. Adding to its biocompatible nature, this hybrid functionalized system can be used for the sustained drug delivery of bioactive molecules or as a tissue engineering scaffold.

## 4. Conclusions

In this work, we demonstrated on two scales (micro and nano) that the functionalization of alginate hydrogel with nanoliposomes does not affect the polymer, while interaction is observed between soft nanoparticles and calcium divalent cations. The incorporation of nanoliposomes in GelMA promotes polymer–nanoparticles electrostatic interactions. These interactions also occur in the polymer-composite hydrogel. When the nanoparticles are added to the mixture, alginate and liposomes compete to react with the positive charges of GelMA. From this point of view, it is interesting to study these modifications that can improve the mechanical properties of the hydrogels. As shown recently [30], nanofunctionalized IPN hydrogels can potentially be used as topical drug delivery systems for wound-healing applications.

## Figures and Tables

**Table 1:** Mean values of zeta potential (mV) of the pure polymers and their mixture before and after the addition of nanoliposomes.

	Without Liposomes	With Liposomes
Alginate	−82.40	−73.87
GelMA	−1.59	−1.26
Alginate + GelMA	−21.83	−21.07
